# Impact of a Community Based Implementation of REACH II Program for Caregivers of Alzheimer's Patients

**DOI:** 10.1371/journal.pone.0089290

**Published:** 2014-02-27

**Authors:** Kristine Lykens, Neda Moayad, Swati Biswas, Carlos Reyes-Ortiz, Karan P. Singh

**Affiliations:** 1 Department of Health Management and Policy, School of Public Health, University of North Texas Health Science Center, Fort Worth, Texas, United States of America; 2 Department of Behavioral and Community Health, School of Public Health, University of North Texas Health Science Center, Fort Worth, Texas, United States of America; 3 Department of Mathematical Sciences, University of Texas at Dallas, Richardson, Texas, United States of America; 4 Geriatrics, Department of Internal Medicine, Oakwood Hospital and Medical Center, Dearborn, Michigan, United States of America; 5 University of Alabama Comprehensive Cancer Center's Biostatistics and Bioinformatics Shared Facility, University of Alabama at Birmingham, Birmingham, Alabama, United States of America; “Mario Negri” Institute for Pharmacological Research, Italy

## Abstract

**Background:**

In 2009 an estimated 5.3 million people in the United States were afflicted with Alzheimer's disease, a degenerative form of dementia. The impact of this disease is not limited to the patient but also has significant impact on the lives and health of their family caregivers. The Resources for Enhancing Alzheimer's Caregiver Health (REACH II) program was developed and tested in clinical studies. The REACH II program is now being delivered by community agencies in several locations. This study examines the impact of the REACH II program on caregiver lives and health in a city in north Texas.

**Study design:**

Family caregivers of Alzheimer's patients were assessed using an instrument covering the multi-item domains of Caregiver Burden, Depression, Self-Care, and Social Support upon enrollment in the program and at the completion of the 6 month intervention. The domain scores were analyzed using a multivariate paired t-test and Bonferroni confidence interval for the differences in pre- and post-service domain scores.

**Results:**

A total of 494 families were enrolled in the program during the period January 1, 2011 through June 30, 2012. Of these families 177 completed the 6 month program and have pre – and post service domain scores. The median age for the caregivers was 62 years. The domain scores for Depression and Caregiver Burden demonstrated statistically significant improvements upon program completion.

**Conclusion:**

The REACH II intervention was successfully implemented by a community agency with comparable impacts to those of the clinical trial warranting wider scale implementation.

## Introduction

In 2009 an estimated 5.3 million people in the United States were afflicted with Alzheimer's Disease (AD), a degenerative form of dementia which begins with memory loss and leads to disorientation, impaired judgment, behavioral changes, and difficulties in speaking, walking, and swallowing in later stages. As Americans age, the number of persons with this condition are anticipated to rise to 11 to 16 million by 2050 [1]. Due to the degenerative nature of this condition, impact of this disease is not limited to the patient but also has significant impact on the lives and health of their family and community caregivers. Caregivers have described feelings of being stressed, overwhelmed, and depressed and lacking emotional and social support, and reported reductions or termination of employment because of their caregiving responsibilities [2].

In order to alleviate this burden on Alzheimer 's disease caregivers and maintain their ability to care for the patient, several interventions have been developed and tested [3]. One of these interventions, Resources for Enhancing Alzheimer's Caregivers Health II (REACH II) was funded in the Tarrant County, Texas (Fort Worth area) by the United Way of Tarrant County in 2010. The authors were contracted to conduct a small scale evaluation of in the REACH II intervention delivered by the Alzheimer's Association of North Texas. In this paper, we examine whether the positive impacts found in the original implementation of the REACH II intervention, which was in a controlled clinical setting could be replicated when the intervention was translated into a community setting.

### Caregiver Burden

Millions of adults in the US are afflicted with Alzheimer's disease (AD) type dementia and majority of them continue to live in the community. According to Schubert et al. [4], 75% of the care for these patients is provided by their family and friends. It is widely acknowledged that caregiving is stressful and perhaps the assertion can be made that caring for AD patients is an even more stressful endeavor due to the care-recipient's emotional, cognitive and behavioral problems such as aggression, agitation, confusion, and nighttime wondering, and the progressive needed assistance with activities of daily living [5]. Indeed, more than 80% of AD caregivers report high level of stress and nearly half report suffering from depression [6]. Moreover, in a meta-analysis, Vitaliano et al. [7] found that caregivers have 23% higher levels of stress hormones and the level of their anti-bodies are 15% lower than non-caregivers.

Providing care to elder family members can at times come at a great cost to the caregiver. Caregivers of AD patients invest significant time, energy, and money, and in many cases over a long period of time, which involves exhausting tasks leading to high levels of burn-out symptoms [6]. Depression along with burn-out symptoms, poor self-rated health, highly perceived stress and lower levels of life satisfaction are factors which affect the health of caregivers by being less likely to engage in preventive health behaviors [4], [8], [9]. As a consequence, caregivers have a meaningful risk for adverse physical health such as serious illness, increased emergency department use and hospitalization, and increased risk of mortality [4], [8]. Caregivers' cumulative stress is also associated with increased nursing home placement or institutionalization and hospitalization of the patient with AD [10]. Thus, caring for a patient with AD leads to undermining health and well-being of both the patient and the caregiver. Hence the implication is that there is a need for interventions that treat both patients and caregivers.

### REACH

The Resources for Enhancing Alzheimer's Caregiver Health (REACH) study was funded by the National Institute on Aging and the National Institute of Nursing Research as a multisite research program. It aimed to test REACH as an effective caregiver intervention, and it was performed in 2 phases. The first phase, REACH I, tested different interventions at 6 U.S. sites (Birmingham, Boston, Philadelphia, Memphis, Miami and Palo Alto; N = 1222 pairs of caregivers and recipients), in order to test various psychosocial interventions and their impact on the health and wellbeing of family caregivers of persons with AD dementia [11]. REACH Investigators assert that study of care giving among minority families has been neglected, and they placed a special emphasis on inclusion of African American and Hispanic caregivers in the REACH intervention. Hence the intervention at each site was tailored to meet racial and ethnic needs of majority and minority populations [12]. The findings of REACH I indicated that active training such as engagement of caregivers in skills training, role playing, and interactive practice were more successful in reducing caregiver burden, compared with more passive methods, such as providing information by giving only educational materials [13], [14]. The findings from REACH I guided the design of REACH II intervention.

REACH II was conducted as a multi-ethnic, multi-site, randomized clinical trial (intervention vs. control group) to reduce burden and depression of caregivers of AD patients [15]. The REACH II intervention included multiple strategies such as providing information to the caregivers, didactic instructions, role playing, stress management techniques, problem solving, skills training and telephone support groups. These interventions were introduced to the caregivers in order to reduce the risk in 5 target areas: depression, burden, self-care/healthy behaviors, social support, and problem behaviors. The intervention provided caregivers with education, skills to manage their care recipient's troublesome behavior, social support, strategies to reframe negative emotional responses and strategies to improve health behavior and stress management [16]. The intervention was delivered by certified interventionists in English and Spanish, over a 6 month period of time, and included 12 sessions [9 in-home, and 3 telephone sessions], and five structured telephone support group sessions [16]. To deliver the intervention, the participating caregivers were given a resource notebook, educational materials and a telephone that adequately supported conference calls. Reach Investigators found that active treatments were more effective than receiving only educational materials in reducing caregiver burden (better self-rated health, sleep quality, physical health and emotional health) and their depression [15], [16].

### Program Implementation

The United Way of Tarrant County contracted with the Alzheimer's Association to implement the REACH II program as a component of support services to Alzheimer families. The Alzheimer's Association Caregiver Education and Counseling REACH II staff consisted of two dementia care specialists (counselors), who received training in the REACH II program implementation. The counselors were trained during a two day workshop offered by a co-investigator of the original REACH development and implementation team. The workshop included training on the content of the resource book, role playing, and discussion of proper responses to various situations raised by caregivers.

The REACH II counselors assessed the caregivers at home and followed up in-person and by telephone counseling, covering topics including home safety, feelings and stress management, behavioral skills training, and provided additional resources in conjunction with material from caregiver notebooks provided by the REACH II program which were tailored to specific needs of the family. The resource notebook was used when the counselors visited or contacted the caregivers by phone. The notebook was composed of sections covering each of the areas addressing the quality of life indicators. The sections included pictures and information with particular attention to the needs identified by the caregivers as their priorities. The REACH II staff made modifications in the delivery of REACH II services to accommodate program implementation in the community setting. One example was that in order to resolve communication barriers with families by changing terminology in interactions substituted “dementia care specialists” for “counselors”. A professionally printed caregiver notebook in Spanish was developed based on a former program in Pittsburgh, PA, to enable program delivery to Hispanic families.

Referral sources to the program included a 24/7 telephone helpline, support groups, case managers, the Aging & Disabilities Resource Center (ARDC), partnering agencies, and home health agencies were utilized to recruit program participants.

A questionnaire developed by the REACH II study was administered to the caregivers of Alzheimer's patients who received REACH II services at the beginning of services and was repeated at the time when services were completed. The intake process was developed and implemented which entailed an initial telephone contact, the first home visit and assessment, followed by additional home visits and or phone calls based on the caregiver needs as established by the assessment tool.

Families included in this analysis were served by the program during the period January 1, 2011 through June 30, 2012. A total of 494 families were served during this period. For caregivers whose loved ones passed away prior to the completion of the 6-month REACH program the relevant parts of the Quality of

Life questionnaire were completed and renamed bereavement battery. This led to improved data capture of services delivered by mainly including the depression and self-care sections.

## Methods

### Variables

The variables recorded for each client include demographic characteristics (race/ethnicity, gender, and age), start and end dates of service, reason for service termination, and pre- and post-service scores for the four domains – Depression, Caregiver Burden, Self-Care, and Social Support. The domains scores were obtained through a sixteen item risk appraisal instrument developed for the REACH II program administered upon intake to and completion of the program [17]. These were calculated as follows. Each domain had several questions. For each question, the client had to choose one of the several possible responses. For calculating the overall scores for each domain, we assigned each type of response a numerical value. Table 1 shows the Domain measures and Range of Scores for each Domain

**Table 1:** REACH II Domains, Measures and Domain Score Ranges.

Domain:	Measures:	Domain Score Range
Caregiver Burden	12 Item Risk Assessment of Stress or Difficulty with Caregiver Tasks in Assisting Care Recipient	0–48
Depression	Center for Epidemiological Studies Depression Scale OR 10 item instrument of feeling sad, depressed or angry Sometimes or more often	0–40
Self-Care	12 item Risk Assessment of Caregiver missing physician appointments, decrease in physical activity, sleeping or nutrition problem	0–12
Social Support	10 item Risk Assessment of availability of someone to talk to or assist with caregiving, feeling isolated	0–50

### Descriptive Statistics/Graphs

We first summarized the demographic characteristics of the study sample. We also classified the clients by their reasons for service termination. As not everyone completed the program, for each demographic category, we found the percentage that completed the program. Finally we compared the pre- and post-service domain scores for clients who had both of these scores available (these scores were not available for all clients completing the program)

### Inference

For formal tests on comparison of pre- and post-service scores, we used paired t-test for each domain. As the four domains are related, we also carried out multivariate paired t-test to test if there are differences between pre- and post-service scores over all domains. As this overall test showed significance, we followed it up by 95% Bonferroni confidence intervals (CIs) for differences between pre and post-service scores for each domain. If a CI for difference does not include the null value of 0, then it gives evidence that the difference between pre- and post-service scores for that domain is statistically significant.

The study participants did not sign a consent letter per se. The participants were recipients of program services being evaluated by the researchers. As such they signed a form given permission for their data to be shared with the Dallas Fort Worth Hospital Council Foundation. This Foundation matches their data with hospital data and provided a de-identified file to the evaluators. This procedure was approved by the University of North Texas Health Science Center (UNTHSC) Institutional Review Board. The study was also approved by the UNTHSC Institutional Review Board.

## Results

We had a total of 494 clients in the sample. The demographic characteristics are shown in Table 2. The majority of them were female and white. The mean and median ages were both about 62 years and the first (25th percentile) and third (75th percentile) quartiles were 53 and 74 years. A total of 177 clients completed the program. The percentage completing the program was about the same for both males and females. However, a higher percentage of Blacks and Hispanics completed the program compared to Whites. Although there were only 3 Asian clients, two of them completed the program which represented the highest proportion among all race/ethnicity categories.

**Table 2:** Demographic Characteristics of the Study Sample and those Completing the Program.

	N (%)	# Completing Program (% of N)
**Gender**		
Male	115 (23.28)	43 (37.39)
Female	379 (76.72)	134 (35.35)
**Race**		
White	329 (66.60)	103 (31.31)
Black	108 (21.86)	47 (43.52)
Hispanic	53 (10.73)	25 (47.17)
Asian	3 (0.61)	2 (66.67)
Not Reported	1 (0.20)	0 (0.00)
**Age**: Mean (SD)	62.49 (13.57)	63 (SD = 13.36)

**Figure 1:**
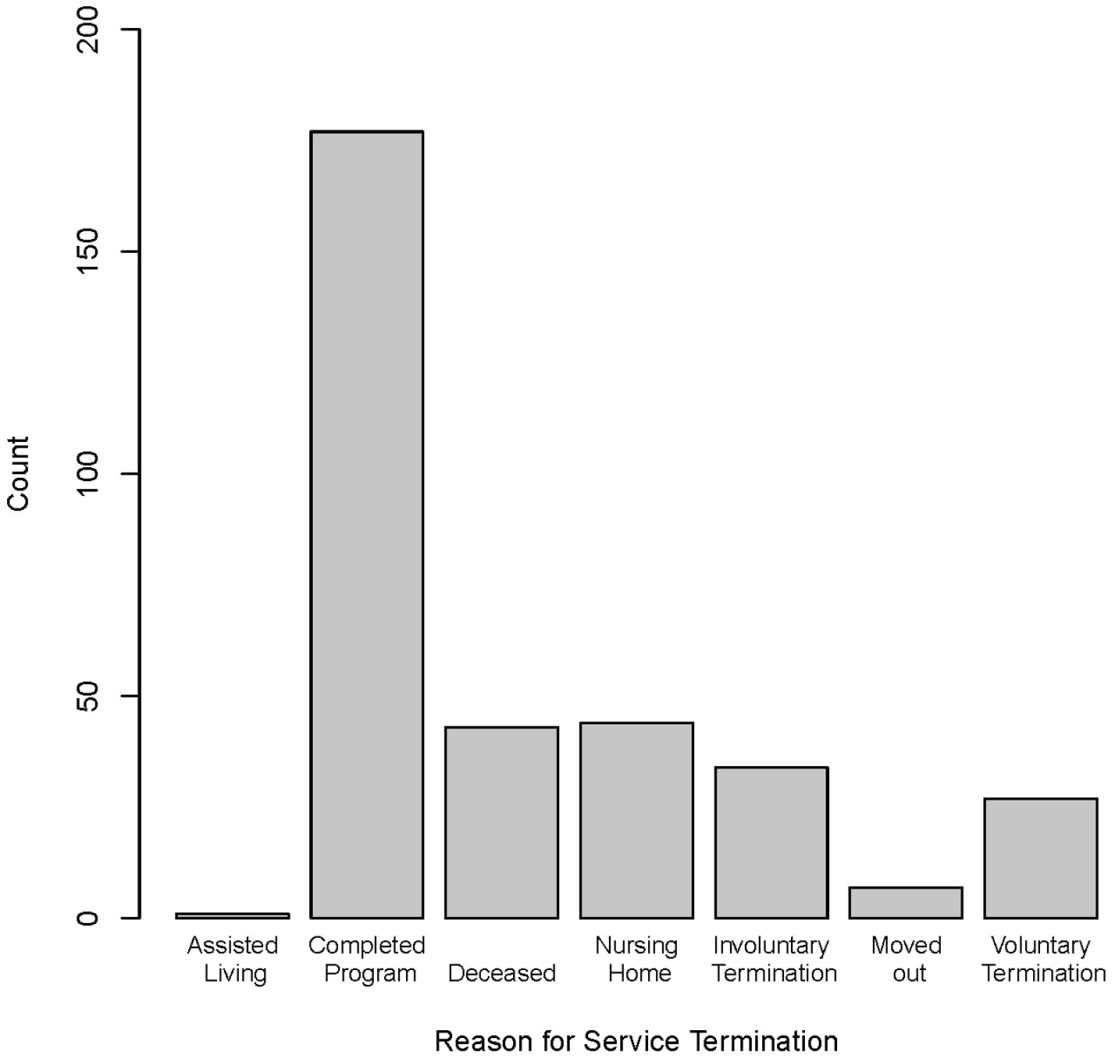
Reason for Termination of Service.

Figure 1 shows the distribution for the reason for service termination. This was known for 333 clients, who had an exit date recorded. Of these clients, similar numbers of the Alzheimer's patients died in this period, got admitted into nursing home, or voluntarily or involuntarily terminated the program. Very few clients moved out of the service area.

**Figure 2:**
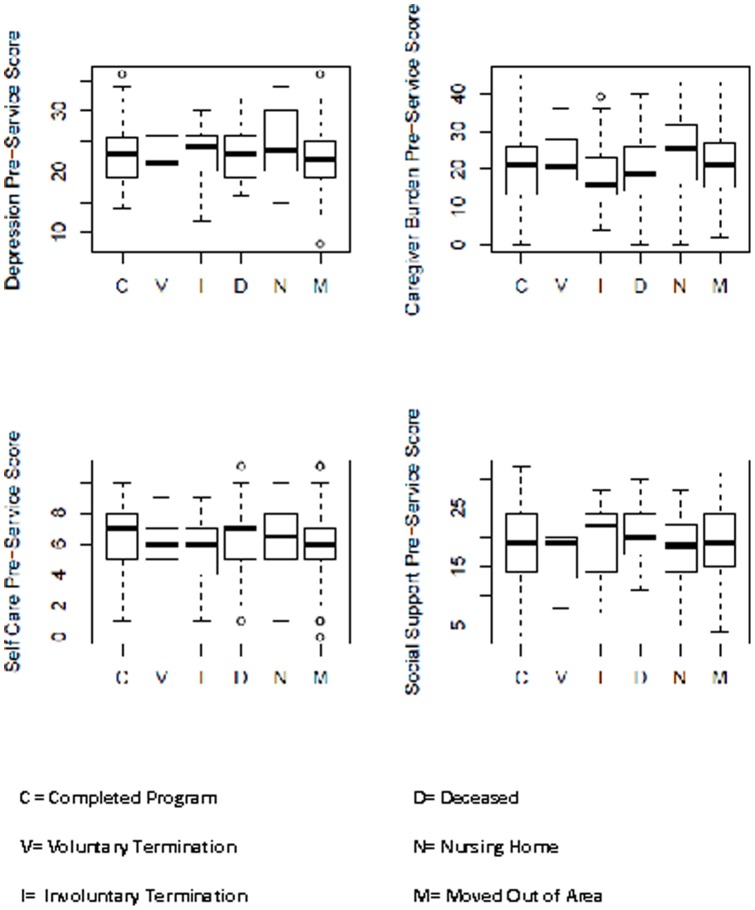
Pre-Service Distribution of Domain Scores by Reason for Termination

Figure 2 presents the distribution of Domain Scores by Reason for Termination of Service. The boxplots contain the median scores in the bold lines within the boxes. The boxes outline the 25^th^ to 75^th^ percentiles of the distribution of scores and the connected lines show the full range of scores for the domain. As shown in this figure the Pre-Service scores were similar among the clients who terminated for the various reasons. Only for the Self Care domain were the scores of those who completed the program higher than those who terminated services. Also of note is that the Caregiver Burden score was highest for those clients who were placed in nursing homes.

**Figure 3:**
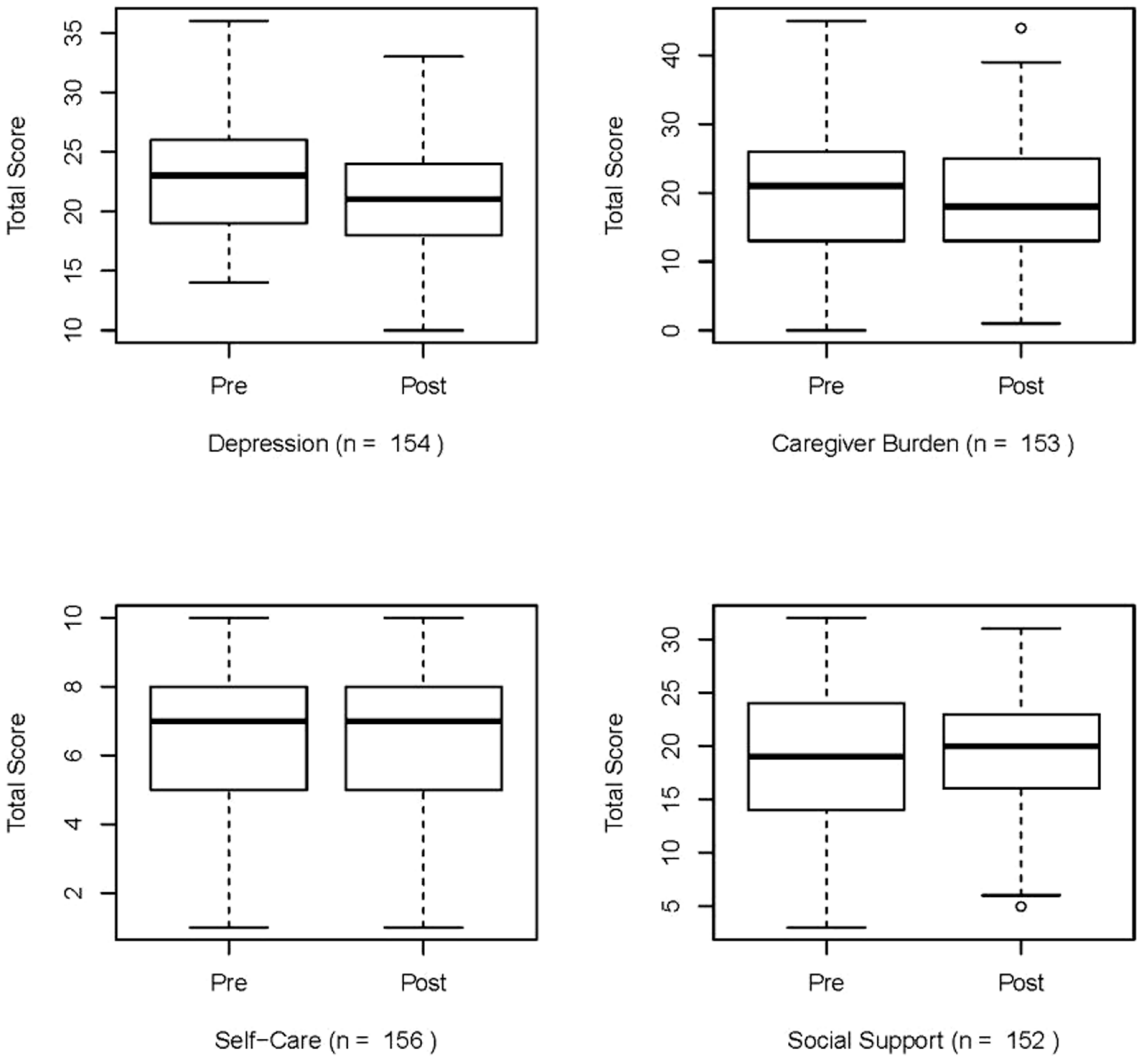
Side-by-side boxplots of Pre- and Post-Service scores for the four domains.

Table 3 and Figure 3 show the pre- and post-service scores for each domain. Note that *n* varies slightly from one domain to other as some clients chose not to respond to some questions in one or more domain. In that case, the total score for that domain for that client cannot be determined. All of the domains showed some improvement between the pre and post scores.

**Table 3:** Comparison of Pre- and Post-Service Domain Scores: Mean (SD) and P-values using the paired t-test.

Domain	N	Pre	Post	P-value
Depression	154	22.88 (4.49)	21.39 (4.19)	<0.0001
Caregiver Burden	153	20.31 (8.92)	18.65 (8.94)	0.025
Self-Care	156	6.33 (1.93)	6.58 (1.76)	0.108
Social Support	152	19.09 (6.09)	19.41 (5.47)	0.495

. Also see Figure 2 for comparison of full pre- and post-service scores distributions. N is the number of subjects who answered all pre- and post-service questions in respective domains. The scores for all questions in a domain were added (with proper weighting) to obtain the score for that domain.

The boxplots of Pre and Post Service scores in Figure 3 show the median scores in the bold line in the center of the plot, the 25^th^ to 75^th^ percentiles within the box, and the full range of scores in the connected lines. They are intended to show the distribution of scores for each domain.

Table 3 shows the statistical analysis of pre-post domain scores. Specifically, the domain scores for Depression and Caregiver Burden reduced post-service compared to pre-service, and these changes were statistically significant with the results for the Depression domain being highly significant. The scores for Self-Care and Social Support increased slightly post-service, which is in the correct direction; however, the results were not statistically significant. The multivariate paired t-test gave similar result (p-value<0.01) showing differences between pre- and post-service scores over all domains considered together. We followed it up with Bonferroni CIs for the differences in pre- and post-service scores for the four domains. They were for Depression: (−2.4, −0.52), Caregiver Burden: (−3.84, −0.19), Self-Care: (−0.16, 0.62), and Social Support: (−0.78, 1.65). As the CIs for Depression and Caregiver Burden do not include 0, we conclude that the differences in pre- and post-service scores for these domains are statistically significant, consistent with results from the univariate paired t-tests.

## Discussion

Almost 10 million family members, friends, and neighbors provided care to Alzheimer's patients in 2005 without payment [1]. Literature has documented that providing this care has significant impacts on the caregiver, which include stress, depression, reduced immune function, hypertension, and heart disease, loss of time at employment, loss of employment and thus income, and out-of-pocket expenses [1], [4]. With one in eight persons over the age of 65 having Alzheimer's Disease or other dementias (5.1 million persons in 2008) and an additional 454,000 estimated new cases per year as of 2010 [1] assisting these caregivers with their responsibilities and coping capabilities is an important part of any strategy to accommodate the aging of the U.S. population.

The Resources for Enhancing Alzheimer's Caregiver Health (REACH) was developed through a study funded by the National Institute of Aging and the National Institute of Nursing Research from which the REACH intervention was selected as an effective intervention to assist caregivers of Alzheimer's disease [13]. The REACH II intervention was further refined to account for different levels of need [15], [16]. This development and testing of the REACH and REACH II interventions were conducted at clinical sites. This study furthers the knowledge regarding the impact of REACH II when it is delivered in a community setting.

Our study has demonstrated that similar proportions (male 37% and female 35%) of caregivers with their Alzheimer's patients complete the program or terminated the program due to the death of the Alzheimer's patient in similar proportions. White caregivers had a lower proportion (31%) completing or terminating the program due to death of the Alzheimer's patient than did Black (44%) or Hispanic (47%) caregivers. This finding is consistent with the earlier REACH II clinical implementation for Hispanics but in our study the proportion of Black caregivers completing was higher than for Whites whereas the opposite was true in the original study [16]. The number of Asian caregivers in our study (3) was too small to reach conclusions. The mean age of the caregivers in our study was 62 years. Thus the caregivers were advancing in age which may cause their caregiving tasks to be more burdensome. A study of institutional placement of persons with dementia had an even greater mean age for the caregivers of 66 years old. This age distribution is likely to continue or increase in years as the numbers of people living into their 80s continues to rise [1]. With increasing age of caregivers the negative consequences of the caregiving role most likely will increase [2].

Of the four caregiver domains which were measured in our study, significant improvements were found for Depression and Caregiver Burden. The domain scores for Self Care and Social Support demonstrated improvement but the changes were not statistically significant. These findings are consistent with the original clinical REACH II study [16]. A study by Powers et al. [18] demonstrated that coping strategies for depression due to caregiving for Alzheimer's patients has become fairly stable over an extended period of time. Thus the REACH II intervention has the potential to improve the quality of life of Alzheimer's caregivers over an extended period beyond the pre-post period we observed. The Caregiver Burden domain which also demonstrated significant improvement in this study measures caregiver feelings of stress and also their good feelings associated with providing assistance to the Alzheimer's patient. This finding is also consistent with the clinically based REACH II study [16]. It suggests that this intervention adds to the confidence the caregiver has in their responsibilities and thus a reduction in the stress related to them.

Limitations of this study are related to the “real world” implementation which provided the REACH II services by a community provider (Alzheimer's Association, North Texas Chapter) with funding from the regional United Way organization. All caregivers who enrolled in the program received the REACH II intervention with no randomization to a control group. We believe that withholding of services could not be ethically justified due to the finding from the original implementation that caregivers significantly benefited from the services when compared to a control group. Delivery of services by a community organization through local funding was not sufficient to support a large array of measurement instruments and no “post” measures were available from caregivers who voluntarily or involuntarily terminated from the program or moved out of the service area.

## Conclusions

Earlier studies of the REACH II intervention have established that this program can improve the quality of life of caregivers of Alzheimer's patients [16] and accomplish this in a cost-effective manner [19]. In a White Paper sponsored by the Alliance for Aging Research [3] numerous effective interventions were identified including REACH II. This paper, however, identifies a lack of funding for translational studies of these interventions in community settings.

Through the United Way of Tarrant County support for a small scale evaluation of multiple programs funded as part of their Healthy Aging and Independent Living initiative, we were able to establish that the REACH II intervention could be successfully implemented in a community setting with outcomes comparable to the original REACH II clinically based study. These findings support a wider scale implementation of the REACH II intervention in community settings to help address the growing demands on family and community caregivers of Alzheimer's patients in the coming decades as the U.S. population continues to age and acquire this condition. Financial support for these services will continue to be a challenge as resources available at the community level are constrained. Policymakers should consider the value of these services as part of their efforts to keep elderly dementia patients in their homes and communities.
